# Does Subretinal Fluid Influence Choroidal Thickness (ChT) and Structure in Preeclampsia with Serous Retinal Detachment? Comment on Fukui et al. Changes in Choroidal Thickness and Structure in Preeclampsia with Serous Retinal Detachment. *J. Clin. Med.* 2023, *12*, 609

**DOI:** 10.3390/jcm12093273

**Published:** 2023-05-04

**Authors:** Andrea Valerio Marino, Aniello La Marca, Martina De Luca, Eleonora D’Aniello, Marco Gioia

**Affiliations:** Department of Medicine, Surgery and Dentistry “Scuola Medica Salernitana”, University of Salerno, Baronissi, 84100 Salerno, Italy

We read with great interest the article by Fukui A et al. concerning the “Changes in Choroidal Thickness (ChT) and Structure in Preeclampsia with Serous Retinal Detachment” [1].

We really appreciated this paper because it focuses on visual impairments during pregnancy.

These types of studies are always very interesting, but we would like to make some comments.

In recent years, ChT measurements have been used to try to explain several conditions [2,3,4].

ChT is axial length related, and according to the literature, it is thicker in shorter eyes and thinner in longer ones. In this study, it was not evaluated; therefore, the ChT of different patients is not comparable and their variations are not reliable. 

We are aware that axial length measurements need some corrections, but they are precise enough for such an aim [5,6].

In the case of macular edema, the exudation in the subretinal space makes it difficult to identify the right point to place the marker for the measurement because of the disappearance of the foveal depression [7]. In this paper, it seems that the follow-up setting was not used to match the images, as it appears from the dissimilar appearance of the optic nerve sheath, so the measurements of ChT cannot be perfectly comparable.

Finally, in our opinion, CVI has a limitation because it can be influenced by the so-called blooming effect. In particular, stromal and luminal areas are affected by the setting of signal amplification; therefore, when high signal amplification is used, the image will appear brighter and the amount of white pixels will be greater, and the opposite will happen using a low setting. This artifact, which is present in the echographic examinations [8,9], also seems to be present in the case of OCT. We are afraid that this effect could also influence the binarization utilized in the CVI evaluation, increasing the low reflective areas, considered to be the luminal ones, when the amplification is low, and reducing them when the amplification is higher.

## Summary Statement

Standardized measurements are needed to obtain reliable data; therefore, the aim of this letter is to clarify some points.
